# The sucrose–trehalose 6-phosphate nexus: what’s next for plant vigour and productivity?

**DOI:** 10.1093/jxb/eraf484

**Published:** 2026-01-12

**Authors:** Brian Ayre

**Affiliations:** Department of Biological Sciences and BioDiscovery Institute, University of North Texas, Denton, TX 76201, USA

**Keywords:** Carbon partitioning, phloem transport, sucrose, trehalose 6-phosphate

## Abstract

This article comments on:

**Annunziata MG, Feil R, Lohse M, Figueroa CM, Hartman MD, Esmailpour M, Nikoloski Z, Koehl K, Stitt M, Lunn JE, Fichtner F.** 2026. The sucrose–trehalose 6-phosphate nexus is conserved in flowering plants with different phloem loading and carbon storage strategies. Journal of Experimental Botany **77**, 578–591. https://doi.org/10.1093/jxb/eraf401

This article comments on:

**Annunziata MG, Feil R, Lohse M, Figueroa CM, Hartman MD, Esmailpour M, Nikoloski Z, Koehl K, Stitt M, Lunn JE, Fichtner F.** 2026. The sucrose–trehalose 6-phosphate nexus is conserved in flowering plants with different phloem loading and carbon storage strategies. Journal of Experimental Botany **77**, 578–591. https://doi.org/10.1093/jxb/eraf401

**The sucrose–trehalose 6-phosphate nexus model states that trehalose 6-phosphate (Tre6P) is both a signal for sucrose (Suc) status and a negative feedback regulator that helps maintain Suc levels within an optimal range. The link between Suc and Tre6P is best established in Arabidopsis and other plants that transport primarily Suc, but angiosperms have evolved multiple phloem loading strategies and use alternative sugars. Annunziata *et al.* (2025) find conservation of the Suc–Tre6P nexus across angiosperms despite different sugars being transported, different mechanisms for phloem loading, and different photoassimilate storage strategies through day/night (diel) cycles. Tre6P as a universal signalling metabolite has implications for increasing crop vigour and productivity.**

It is reasonably intuitive that plant growth and productivity is coupled to the availability and transport of photoassimilated carbon from source organs (regions of net production) to sink organs (regions of net demand). How plants monitor their reduced carbon levels and use this information to control partitioning through the phloem is more enigmatic. Research from many laboratories over several decades has shown that Tre6P is an important signalling metabolite involved in regulating growth and development in response to carbon availability, specifically Suc (O’Hara *et al.*, 2013; Yadav *et al.*, 2014; Figueroa and Lunn, 2016; Fichtner and Lunn, 2021). The Suc–Tre6P nexus model argues that Tre6P is both a signal for Suc status and a feedback regulator that helps maintain Suc levels within an optimal range (Yadav *et al.*, 2014; Fichtner and Lunn, 2021). The link between Suc and Tre6P is best established in Arabidopsis (*Arabidopsis thaliana*) and other plants, including several important crops, that transport primarily Suc. However, although Suc is transported in all plants, angiosperm lineages have also evolved different phloem loading strategies and use alternative sugars (Rennie and Turgeon, 2009; Zhang and Turgeon, 2018). Annunziata *et al.* (2025) questioned if Tre6P is a universal signalling metabolite, irrespective of loading mechanism and transport sugar, and find support for conservation of the Suc–Tre6P nexus despite different sugars being transported, different mechanisms for phloem loading, and different photoassimilate storage strategies through day/night (diel) cycles (Annunziata *et al.*, 2025).

Plants need to carefully allocate photoassimilate in source organs between general metabolism, storage reserves, and transport to maintain necessary pools throughout diel cycles. Export of photoassimilate from mature leaves initiates with phloem loading, which can be broadly classified as phloem loading from the apoplast or phloem loading through the symplast (i.e., apoplastic phloem loading and symplastic phloem loading, respectively), and as energized or passive (Rennie and Turgeon, 2009; Ayre and Turgeon, 2018). One mechanism is energized phloem loading from the apoplast, which is best studied and may be most prevalent, especially among crop plants. Energized apoplastic loading involves Suc efflux from mesophyll cells surrounding the phloem via SUGAR WILL EVENTUALLY BE EXPORTED TRANSPORTERS (SWEETs), followed by uptake into phloem companion cells by SUC TRANSPORTERS (SUTs) energized by the proton motive force. A second mechanism is energized symplastic phloem loading, which occurs via a polymer trap mechanism: Suc diffuses from phloem parenchyma cells through highly branched plasmodesmata into specialized companion cells (called intermediary cells) and is then converted to raffinose family oligosaccharides that accumulate because they are too large to diffuse back towards the mesophyll. A third mechanism is passive symplastic phloem loading: Suc concentration is highest in the mesophyll and diffuses down a gradient into the companion cells through ‘regular’, unbranched plasmodesmata. Also, some plants use sugar alcohols in addition to Suc for energized apoplastic phloem loading or passive symplastic loading. Transitory storage of photoassimilated carbon during day and subsequent re-mobilization for use and transport at night also varies across angiosperms. Accumulation of transitory starch in chloroplasts is most common and most well studied, but some lineages store significant quantities of Suc or organic acids, principally malate, in vacuoles.

Tre6P in source leaves of Arabidopsis and other plants with apoplastic phloem loading has a duality that helps modulate feast/famine responses (Figueroa and Lunn, 2016). If Suc accumulates in source leaves because demand for transport is low, Tre6P is proposed to divert photoassimilate away from Suc and towards organic acids and amino acids during the day and reduce the remobilization of transitory starch reserves at night (Martins *et al.*, 2013; Figueroa *et al.*, 2016). Conversely, if Suc levels are low and insufficient for transport needs, Tre6P promotes Suc production during the day and starch degradation at night to increase those levels. In sink organs, Tre6P promotes Suc utilization and growth in part by reducing activity of the protein kinase complex, SUC-NON-FERMENTING1-RELATED KINASE1 (SnRK1), which is an inhibitor of growth (Baena-González *et al.*, 2007; Zhang *et al.*, 2009). Tre6P binds directly to the SnRK1α catalytic subunit and lowers the activity of the SnRK1 complex (Zhai *et al.*, 2018) and Tre6P inhibition of SnRK1 also leads to changes in gene expression (O’Hara *et al.*, 2013; Fichtner and Lunn, 2021). Considering the influence of Tre6P in both source and sink organs of plants that utilize apoplastic phloem loading, it is noteworthy that elevated Tre6P levels stimulate the expression of *SWEET* Suc efflux carriers in vascular tissues (Oszvald *et al.*, 2018; Fichtner *et al.*, 2021). More *SWEET* expression in the vascular tissues of source leaves may promote Suc efflux to the apoplast in preparation for SUT-mediated phloem loading, and higher expression in sink organs may promote phloem unloading to supply growth and metabolism.

While there is compelling evidence to link Tre6P with control of Suc levels, metabolism, and transport in both source and sink organs of plants with apoplastic phloem loading, questions remain if this regulation extends to other transport systems. To address the universality of the Suc–Tre6P nexus model, Annunziata *et al.* (2025) carefully chose angiosperms representing different transport and storage systems, and correlated Tre6P levels with other metabolites. Arabidopsis is a model species for studying apoplastic phloem loading and starch storage during the day and consumption at night. Wheat (*Triticum aestivum*) also loads Suc from the apoplast into the phloem, but Suc in the vacuole is the prominent storage carbohydrate in leaves. Strawberry (*Fragaria × ananassa*) and alchemilla (*Alchemilla mollis*) are members of the Rosaceae that load passively through the symplast. Plantago (*Plantago major*) loads Suc and the sugar alcohol sorbitol into the phloem by energized uptake from the apoplast. Melon (*Cucumis melo*) transports raffinose family oligosaccharides by energized loading through the symplast with polymer trapping. Although Tre6P and Suc levels varied substantially among the species, all species showed a correlation between Tre6P and Suc transport pools in leaves when measured across the entire diel cycle. When day and night periods were measured separately, all species showed correlations during the day, and some species had even stronger correlations during the night. These results imply a link between Tre6P and Suc transport pools whether the Suc is derived from photosynthesis or from re-mobilized storage pools. Melon showed a correlation between Tre6P and the transport sugars Suc and raffinose, but not stachyose, throughout the diel cycle. Plantago showed a correlation between Tre6P and Suc during the day but not during the night, and a link between Tre6P and sorbitol was not observed.

In more detailed correlation-based network analysis, conservation of a link between Tre6P and Suc levels across angiosperms was further supported by Gaussian graphical models that represented 22 core metabolites as nodes, and positive and negative associations represented as edges. A community detection algorithm was used to identify functional modules within the network. Considering the metabolic variability in the species analyzed, it is no surprise that species-specific network differences were identified, but despite this, the link between Tre6P and Suc was maintained across five of the six species, with sorbitol-transporting plantago being the exception.

The widespread conservation of the Suc–Tre6P nexus across angiosperms—largely independent of transient storage and phloem transport strategies—has implications for crop productivity. Since an association between Tre6P levels and plant vigour was first identified, increasing Tre6P levels through breeding or biotechnology has been a goal. In grain crops, and likely other crops as well, it is proposed that domestication has selected Tre6P signalling to modulate photoassimilate partitioning from source to sink, and also enhance sink-specific allocation, including grain filling, as well as other domestication traits (O’Hara *et al.*, 2013; Paul *et al.*, 2020). Biotechnology through transgenic or gene-editing approaches holds promise to further enhance beneficial domestication traits. However, bringing gains observed in controlled environments to production scale may prove difficult because beneficial Tre6P signalling shows both spatial and temporal specificity in target tissues, which may be difficult to emulate in open agricultural fields. In addition, each crop needs to be created individually, which is laborious and time consuming, and public resistance towards genetically modified organisms in general may also hinder gains through biotechnology (Paul *et al.*, 2020).

Recently, Tre6P levels were increased in wheat by applying a membrane permeant and sunlight-activated compound, 6-O-Bis-(4,5-dimethoxy-2-nitrobenzyloxyphosphoryl)-D-trehalose (DMNB-Tre6P) (Griffiths *et al.*, 2016; Griffiths *et al.*, 2025). In controlled environments, application of DMNB-Tre6P increased Tre6P levels *in planta*, and increased wheat yields by up to 18% (Griffiths *et al.*, 2016). In subsequent open field trials over four seasons with four wheat varieties, ‘microdose’ DMNB-Tre6P applications 10 days after anthesis provided an overall average yield increase of 10.4% (Griffiths *et al.*, 2025). The authors of these trials propose that uptake of the precursor and conversion to Tre6P enhances sink capacity in developing seeds through up-regulation of transcription factors and regulatory steps ranging from Suc transport through to starch synthesis. In addition, DMNB-T6P treatment may have coordinated observed increases in flag leaf photosynthesis with enhanced vascular connections in the grain to link heightened source strength with greater sink demand (Griffiths *et al.*, 2025). The work by Annunziata *et al.* (2025) showing that the Suc–Tre6P nexus is broadly conserved across angiosperms is promising evidence that manipulating Tre6P levels by breeding, biotechnology, or agrochemical application could bring productivity gains to the majority of our crop species.
